# A fast assay to gauge for TAA-reactive T cells in PBMCS from patients with pancreatic cancer

**DOI:** 10.1186/2051-1426-2-S3-P25

**Published:** 2014-11-06

**Authors:** Elena B Rangelova, Qingda Meng, Liu zhenjiang, Thomas Poiret, Bartek Jiri, Caroline Verbeke, Ernest Dodoo, Ralf Segersvärd, Markus Maeurer

**Affiliations:** 1 Karolinska Insitutet, Karolinska University Hospital, Stockholm, Sweden; 2 Karolinska Insitutet, Stockholm, Sweden; 3 Dept. of Neurosurgery, Karolinska University Hospital, Stockholm, Sweden

## Purpose

Active cellular therapy (ACT) using *ex-vivo *expanded T cells from patients with cancer, obtained by apheresis, can represent a viable source for anti-cancer directed cellular therapy. We established a T cell expansion protocol using 2 rounds of re-stimulation with TAA peptides along with IL-2, IL-15 and IL-21. In order to gauge the ex-vivo cellular reactivity as well as the potential to successful expand antigen-specific T cells from patients with pancreatic cancer, we established a screening assay using whole-heparin blood, to gauge for TAA reactivity (NY-ESO-1, survivin and mesothelin) and control antigens (EBNA-1, EBNA-3, CMVpp65).

## Methods

Fresh blood samples were obtained from 24 patients with pancreatic cancer and from 6 individuals with pre-malignant lesions and tested for anti-TAA reactivity. T cells were expanded without cytokines, with IL-2 and IL-7, or with IL-2, IL-15 and IL-21 and tested for CD4/8 expansion by flow cytometry and for IFN-gamma production. PBMCs were expanded by cytokines and TAA peptides. CD3, CD4, CD8, CD45RA and CCR7 was determined by flow cytometry and TAA-reactive T cells were identified by ICS (IL-2, TNF, IFN and IL-17).

## Results

We could detect IFN-gamma responses in 90% (27 in 30) in blood samples for mesothelin, 55,3% (16 in 30) for survivin and 43,3% (13 in 30) for NY-ESO-1. Cellular responses could be augmented by adding cytokines, i.e. IL-2 and IL-7 could favored CD4+ T cell proliferation, IL-2, IL-15 and IL-21 favored CD8+ T cell proliferation. TAAs-reactive T cells could be successfully expanded in vitro and exhibited TAA-specific production of IFNgamma and TNFalpha and a CD8+CD45RA-CCR7+ phenotype.

## Conclusion

A TAA-specific WBA (whole blood assay) can be used to gauge the potential for expansion of TAA-reactive T cells in peripheral blood from patients with pancreatic cancer. TAA-reactive T cells can be successfully expanded in IL-2, IL-15 and IL-21 and could represent a viable source for the cellular therapy of patients with pancreatic cancer.

## Consent

Written informed consent was obtained from the patient for publication of this abstract and any accompanying images. A copy of the written consent is available for review by the Editor of this journal.

